# Acute Lymphatic Filariasis Infection in United States Armed Forces Personnel Deployed to the Pacific Area of Operations during World War II Provides Important Lessons for Today

**DOI:** 10.3390/tropicalmed5020063

**Published:** 2020-04-17

**Authors:** Wayne D. Melrose, Peter A. Leggat

**Affiliations:** 1 World Health Organization Collaborating Centre for Vector-Borne and Neglected Tropical Diseases, College of Public Health, Medical and Veterinary Science, James Cook University, Townsville 4811, Australia; peter.leggat@jcu.edu.au; 2 School of Public Health, Faculty of Health Sciences, University of the Witwatersrand, Johannesburg 2001, South Africa

**Keywords:** medical history, military, WW2, lymphatic filariasis, helminth, Pacific

## Abstract

The deployment of United States (US) Armed Forces personnel into the central Pacific islands of Samoa and Tonga, which is highly-endemic for lymphatic filariasis (LF), resulted in thousands of cases of the acute form of this disease and greatly reduced their ability to carry out their mission. The major driving factor for the intensity of transmission was the aggressiveness and efficiency of the *Aedes* species mosquito vectors, especially the day-biting *Ae. Polynesiensis.* The paper reminds us of the danger that tropical diseases can pose for troops sent into endemic areas and constant and careful surveillance that is required to prevent rapid resurgence of *Aedes*-transmitted LF in populations, where the LF elimination program has been successful.

## 1. Introduction

Lymphatic filariasis (LF) is a mosquito-transmitted parasitic disease caused by infection with the filarial worms, *Wuchereria bancrofti*, *Brugia* *malayi* and *Brugia timori.* The adult worms live within the lymphatics and the female worms liberate motile embryonic forms called microfilariae into the peripheral blood. In parts of the world where the LF vectors are *Anopheline, Culex* and *Mansonia* mosquitos that bite mostly at night, microfilariae numbers in the blood peak during the night (nocturnal periodicity). In the central Pacific, where the vectors are *Aedes* mosquitoes that bite mostly during the day, microfilariae numbers peak at midday (diurnal periodicity). The global burden of LF is estimated to be around 70 million and its debilitating manifestations of lymphedema, elephantiasis and hydrocele disable around 36 million making it one of the leading causes of chronic disability [1]. There is also an acute form of LF characterized by fever, lymphangitis, and lymphadenopathy and in males, scrotal inflammation [2]. This form of LF is the main focus of this paper.

In 1997, the 50th World Health Assembly passed a resolution to eliminate LF as a public health problem and, in 1999, the Pacific Program for the Elimination of LF (PacELF) was established to eliminate the disease in the 22 LF-endemic countries in the Pacific region through a strategy of annual rounds of mass drug administration (MDA) [3]. By March 2020, Cook Islands, Kiribati, Marshall Islands, Niue, Palau, Tonga, Vanuatu, Wallis and Futuna have been certified by the World Health Organization (WHO) as having achieved the elimination target and several other countries are completing the data collection required for certification and, after completing LF surveys, the Solomon Islands has been declared to be nonendemic and not requiring an MDA program [3,4,5]. This is an outstanding achievement, but the history of LF in the Pacific area during World War II (WW2), provides important lessons that need to be taken into account to prevent a resurgence in the disease.

## 2. Lymphatic Filariasis in the Pacific Region during WW2

During WW2, LF was a major health issue for the US Armed Forces stationed in filarial-endemic areas of the South Pacific and this has been comprehensively reviewed by Coggeshall [6] and Wartman [7]. Although cases occurred throughout the area, the highest number of cases occurred in the central Pacific, especially in Samoa (then consisting of “Western Samoa” and “American Samoa”) and Tonga. There were 127 cases of LF reported at a field hospital in Samoa over a 4-month period [8] and in one unit stationed in Samoa, 70% of the exposed troops became infected. On Tonga-Tabu Island in Tonga, 532 men were diagnosed with LF in a single year [9].

## 3. The Clinical Features of LF in American Armed Forces Personnel

The very sensitive and specific filarial antigen tests that are now regarded as the “gold standard” for LF infection [1] were not available back then, but the diagnosis was not in doubt. The episodic acute attacks of malaise and fatigue, urticaria, painful inflammation and swelling of the genitalia and lymphangitis of the arms and legs, sometimes precipitated or worsened by heavy exercise, are consistent with acute LF (7,10]. Surprisingly, fever was not commonly reported. Fever is commonly seen in acute LF, which is often called “filarial fever” [10], and the description of “Mumu”, which is what acute LF is called in the Samoan language, stresses fever as an important symptom [6]. In 30% of all cases adult worms were removed by surgery or seen in histological sections [7] but only about 20 cases microfilaraemia were detected [7,9]. This was not surprising because microfilaraemia is very rare in acute LF. The exact reason is not known, but possible explanations are that microfilaria are trapped within inflammatory tissue, or rapidly destroyed by the acute inflammatory reaction once they enter the circulation, that the adult worms are of a single sex, or the adult females are too young to produce microfilariae [1,7,9,10]. It is also possible that low numbers of microfilaria were not detected, because of the poor sensitivity of the blood film method used at the time. Modern practice is to use more sensitive concentration techniques [1]. The most common laboratory finding was eosinophilia [7,11].

An interesting observation is that psychological disturbances were common and recorded by several authors [12,13,14]. Symptoms included depression, difficulty in concentrating, anxiety, insomnia and fearfulness. Some of these symptoms may have been prompted by a fear that they might develop some of the severe chronic pathology of LF such as hydrocele and elephantiasis seen in the local population, and that they would, as one writer puts it, “go home with their scrotum in a wheelbarrow” or suffer a loss of sexual function. Chronic pathology is the result of a long-standing, complex interaction between the parasite and the host’s immune system, and, very importantly, the skin and tissue damaged caused by recurrent bacterial and fungal infections [1]. None of these men remained in the endemic area long enough for this to occur. The prevalence of sexual dysfunction or sterility was no higher in LF patients than in the general population [7].

## 4. How Long Did the Clinical Evidence of LF Persist after Returning from an Endemic Area?

No specific treatment was available for LF at that time and the length of time it took for the signs and symptoms of LF to resolve after returning from and endemic area varied. For most cases, it was 20–30 months and for others it took 3 years [7,15] and a careful evaluation of a group of cases after 15 years showed that around a third of them still suffered from acute attacks. A single case who got LF during his deployment to Samoa still complained of symptoms in 1972 [16], and one of the rare microfilaraemic cases still had microfilaria in his blood after 15 years [17,18].

## 5. Why Did Most of These Cases Occur in the Central Pacific Area?

The reason that Wartman and many of the other authors give, is that that the US Armed Forces personnel were in very close contact with infected local inhabitants in an area that was highly endemic for LF that was transmitted by a day-biting mosquito [7]. In addition, most of the troops’ activities occurred during the day; hence their exposure rate was very high [7]. By contrast, only a small number of LF cases were recorded among the thousands of US Armed Forces personnel stationed in the Melanesian countries of Papua New Guinea, the Solomon Islands and the New Hebrides (now Vanuatu) that were also highly filarial endemic [7]. In this area, the vectors are night biting Anopheline species [1], but troops who were occupying positions and fighting both day and night would be just as exposed as their comrades in the central Pacific. In Tonga, the vectors are the mainly day-biting *Ae. tongae* and *Ae. tabu* [5]. In American Samoa and Samoa, the primary vector is the aggressive and highly efficient day-biting Ae. polynesiensis. Other vectors include Ae. samoanus (night-biting), Ae. tutuilae (night-biting), and Ae. upolensis (day-biting) [19]. *Aedes* species breed prolifically in a wide range of places such as tree holes, leaf axils, water-filled tree stumps, coconut shells and crab holes on beaches, and very importantly, in artificial containers like buckets, cans, bottles and tires, which you would expect to be present in big numbers where troops congregate. [20]. Research shows that *Ae. polynesiensis* is a more efficient LF vector than anophelines, especially when the number of circulating microfilariae is low [21]. All these factors meant that the US Armed Forces personnel on the highly-endemic islands of Samoa and Tonga, often working out in the open and wearing only minimal clothing, were constantly being attacked by large numbers of aggressive, effective and efficient LF vectors. Given these circumstances, it understandable that the number of LF cases were so high. By contrast, there are some other factors that might help explain why LF cases were lower in Melanesia, where LF and malaria are transmitted concomitantly by the same vector. It is extremely likely that the use mosquito repellent, inducing troops to cover up as much skin as possible and, later in the war, the use of Dichlorodiphenyltrichloroethane (or DDT) to combat malaria also reduced LF transmission. There is good evidence for this from the Solomon Islands where a control program against malaria resulted in elimination of LF without any specific intervention against LF.

## 6. So What Lessons Can Be Learned from This WW2 Experience?

Firstly, it is a very good example of how deployments can unexpectedly expose a large number of armed forces personnel to a tropical disease or other infective agent that can have a serious effect on their ability to carry out their mission. Several authors cited above point out that little was known about LF, especially its acute form, and the possible threat to the US Armed Forces personnel prior to them being sent overseas. Tropical diseases can still pose a threat to modern day military forces deployed to areas, where they are endemic, and measures must be taken to mitigate the risk [21].

Secondly, it reminds us of the importance of recognizing the acute form of LF and the need to be aware that it can occur in travelers, expatriate workers, defense force members and others who have a relatively short exposure especially where especially efficient vector mosquitoes are present [2].

Thirdly, it has very important lessons for the LF elimination program in the parts of the Pacific, where *Aedes* species are the vectors. As mentioned in the introduction, Tonga has achieved the LF elimination target [5]. Samoa and American Samoa are making progress but are struggling to meet the target with residual pockets of infection in some areas and in some populations [19]. That should be no surprise given that the main vector is *Ae. polynesiensis.* Even when the LF prevalence is brought down to the elimination target there will need to be constant vigilance against resurgence, because unlike other mosquito species, *Ae. polynesiensis* also has the ability to efficiently transmit LF when population prevalence of the parasite is low and there is a small number of circulating microfilariae [22,23,24]. Lack of prior exposure to LF and the lack of immune resistance undoubtedly played a part in the rapid and widespread acquisition of infection in the US Armed Forces personnel and this is acknowledged by many of the authors cited above. LF protective immunity is still poorly understood and how long it will persist in communities after elimination is achieved is not known. It must be remembered that the goal of the LF program is the elimination of LF as a public health problem. It is not an eradication program, and some active cases could still arise in the future. There is a risk that LF-infected migrants could also re-introduce the parasite, especially in places where *Aedes* is the vector [25]. Will the re-appearance of a small number of LF cases spark the sort of outbreaks in these communities that were seen in the WW2 troops, as the local population, like those US Armed Forces personnel, are now immunologically naïve towards the parasite?
